# DOSAGE study: protocol for a phase III non-inferiority randomised trial investigating dose-reduced chemotherapy for advanced colorectal cancer in older patients

**DOI:** 10.1136/bmjopen-2024-089882

**Published:** 2024-08-13

**Authors:** Joosje C Baltussen, Frederiek van den Bos, Marije Slingerland, Trishika R R Binda, Gerrit-Jan Liefers, Wilbert B van den Hout, Marta Fiocco, Arjan J Verschoor, Marissa Cloos-van Balen, Cynthia Holterhues, Danny Houtsma, Anouk Jochems, Leontine E A M M Spierings, Leti van Bodegom-Vos, Simon P Mooijaart, Hans Gelderblom, Frank M Speetjens, Nienke A de Glas, Johanneke E A Portielje

**Affiliations:** 1 Department of Medical Oncology, Leiden Universitair Medisch Centrum, Leiden, Zuid-Holland, The Netherlands; 2 Department of Gerontology and Geriatrics, Leiden University Medical Center, Leiden, The Netherlands; 3 LUMC Center for Medicine for Older People, Leiden University Medical Centre, Leiden, The Netherlands; 4 Department of Surgery, Leiden University Medical Center, Leiden, The Netherlands; 5 Department of Biomedical Data Sciences, Section of Medical Decision Making, Leiden Universitair Medisch Centrum, Leiden, Zuid-Holland, The Netherlands; 6 Department of Biomedical Data Sciences, Section of Medical Statistics and Bioinformatics, Leiden Universitair Medisch Centrum, Leiden, Zuid-Holland, The Netherlands; 7 Mathematical Institute, Leiden University, Leiden, The Netherlands; 8 Department of Medical Oncology, Reinier de Graaf Hospital, Delft, The Netherlands; 9 Department of Internal Medicine, Groene Hart Ziekenhuis, Gouda, Zuid-Holland, The Netherlands; 10 Department of Medical Oncology, Haga Hospital, Den Haag, The Netherlands; 11 Department of Medical Oncology, Medisch Centrum Haaglanden Westeinde, Den Haag, Zuid-Holland, The Netherlands; 12 Department of Internal Medicine, Alrijne Hospital Leiden, Leiden, Zuid-Holland, The Netherlands

**Keywords:** Randomized Controlled Trial, Gastrointestinal tumours, CHEMOTHERAPY, GERIATRIC MEDICINE, ONCOLOGY, Quality of Life

## Abstract

**Introduction:**

Treating older adults with chemotherapy remains a challenge, given their under-representation in clinical trials and the lack of robust treatment guidelines for this population. Moreover, older patients, especially those with frailty, have an increased risk of developing chemotherapy-related toxicity, resulting in a decreased quality of life (QoL), increased hospitalisations and high healthcare costs. Phase II trials have suggested that upfront dose reduction of chemotherapy can reduce toxicity rates while maintaining efficacy, leading to fewer treatment discontinuations and an improved QoL. The DOSAGE aims to show that upfront dose-reduced chemotherapy in older patients with metastatic colorectal cancer is non-inferior to full-dose treatment in terms of progression-free survival (PFS), with adaption of the treatment plan (monotherapy or doublet chemotherapy) based on expected risk of treatment toxicity.

**Methods and analysis:**

The DOSAGE study is an investigator-initiated phase III, open-label, non-inferiority, randomised controlled trial in patients aged≥70 years with metastatic colorectal cancer eligible for palliative chemotherapy. Based on toxicity risk, assessed using the Geriatric 8 (G8) tool, patients will be stratified to either doublet chemotherapy (fluoropyrimidine with oxaliplatin) or fluoropyrimidine monotherapy. Patients classified as low risk will be randomised between a fluoropyrimidine plus oxaliplatin in either full-dose or with an upfront dose reduction of 25%. Patients classified as high risk will be randomised between fluoropyrimidine monotherapy in either full-dose or with an upfront dose reduction. In the dose-reduced arm, dose escalation after two cycles is allowed. The primary outcome is PFS. Secondary endpoints include grade≥3 toxicity, QoL, physical functioning, number of treatment cycles, dose reductions, hospital admissions, overall survival, cumulative received dosage and cost-effectiveness. Considering a median PFS of 8 months and non-inferiority margin of 8 weeks, we shall include 587 patients. The study will be enrolled in 36 Dutch Hospitals, with enrolment scheduled to start in July 2024. This study will provide new evidence regarding the effect of dose-reduced chemotherapy on survival and treatment outcomes, as well as the use of the G8 to choose between doublet chemotherapy or monotherapy. Results will contribute to a more individualised approach in older patients with metastatic colorectal cancer, potentially leading to improved QoL while maintaining survival benefits.

**Ethics and dissemination:**

This trial has received ethical approval by the ethical committee Leiden Den Haag Delft (P24.018) and will be approved by the Institutional Ethical Committee of the participating institutions. The results will be disseminated in peer-reviewed scientific journals.

**Trial registration number:**

NCT06275958.

**STRENGTHS AND LIMITATIONS OF THIS STUDY**

- The uniqueness of this investigator-initiated trial lies in its design, specifically tailored for older patients.
- The choice between monochemotherapy and doublet chemotherapy will be individualised based on the Geriatric 8 Questionnaire.
- The DOSAGE trial measures meaningful endpoints relevant to older adults, such as quality of life, physical functioning and hospitalisations.
- As recruiting older adults in trials may be challenging, the study will be enrolled in 36 Dutch hospitals, with efforts to keep the participation burden low.
- To reduce the risk of undertreatment in the dose-reduced arm, the dose may be escalated after two cycles if there is good tolerability

## Introduction

 Colorectal cancer is one of the most frequently diagnosed cancers in older adults: 60% of patients with colorectal cancer are aged ≥65 years, with approximately one-third exceeding 75 years.^1^ Among them, half will eventually develop distant metastases or present with metastasised disease at time of diagnosis and will be needing chemotherapy. Yet, pivotal chemotherapy trials included very few older patients, and if included, they were generally very fit.^2^ This strongly limits the evidence base for the treatment of the majority of older adults with an average health status or frailty. Due to this lack of knowledge, there are no Dutch guidelines on how to tailor treatment for older adults with metastatic colorectal cancer, leading to high toxicity rates and unplanned hospitalisations and a reduced quality of life (QoL).^3^,^5^ This makes chemotherapy both less effective (due to early treatment discontinuation) and expensive for society. In addition, data from the Dutch cancer registry have showed no survival improvement for older patients with metastatic colorectal cancer in the past 10 years,^6^ suggesting that only a subset currently benefits from novel treatment options. Since society faces an ageing population as well as concerns of increasing healthcare costs, specific trials targeting this growing population are thus an unmet societal and medical need.

The main challenge in individualising treatment of older adults with metastatic colorectal cancer is the large heterogeneity between patients. While some patients are physically ‘fit’ and have few other concomitant diseases, others may have age-associated problems such as multimorbidity or physical or cognitive impairments, making them ‘frail’. These factors strongly influence the ability to endure chemotherapy.^4 5 7 8^ Older adults with metastatic colorectal cancer are commonly treated with either fluoropyrimidine-based monotherapy, or doublet chemotherapy with a fluoropyrimidine and oxaliplatin.^9^ High toxicity rates are seen in both regimens, but this effect is more pronounced in frail patients and in doublet chemotherapy.^10^ However, Dutch colorectal cancer guidelines do not give specific advice on the selection of older patients for either doublet chemotherapy or monotherapy in relation to risk of frailty or vitality,^11^ resulting in undesirable variations in treatment regimens across hospitals.

The Geriatric 8 (G8) Questionnaire is a geriatric screening tool that detects health deficits in different domains and risk of frailty in older patients.^12^ The tool has been well validated in oncology practice, with various studies demonstrating that older adults who score low on the G8 were more likely to experience chemotherapy-related toxicity compared with those with a normal G8 Score.^7 8 13^ The G8 serves as a simple and easy-to-use risk stratification tool for toxicity, offering a practical alternative to the geriatric assessment (GA), which is much more time-consuming and ideally should be done by trained staff. Additionally, the G8 tool is already widely used in Dutch hospitals, as underscored by a recent study showing that 97% of Dutch hospitals offering colorectal cancer surgery use the G8 for frailty screening,^14^ which will facilitate its implementation. Therefore, the G8 can be used for a tailor-made treatment decision between monotherapy or doublet chemotherapy.

Another potential solution to reduce toxicity risk is to perform upfront dose reduction of chemotherapy. Prior studies demonstrated that upfront dose reduction of chemotherapy in older adults with advanced cancer decreased toxicity rates by 20%–30%, while maintaining efficacy.^15 16^ For example, the phase II NORDIC9 trial compared full-dose S1 monotherapy with upfront dose-reduced S1 with oxaliplatin in 160 older adults with metastatic colorectal cancer judged ‘unfit’ for full-dose treatment by their oncologist.^16^ The study showed that doublet chemotherapy with upfront dose reduction resulted in a better progression-free survival (PFS) (6 months for reduced dosed vs 5 months for full dose), with markedly reduced toxicity rates in the dose-reduced arm (43% vs 62%, respectively). In addition, the GO2 trial compared different levels of dose-reduced capecitabine plus oxaliplatin (CAPOX) in 559 advanced gastro-oesophageal cancer patients deemed unfit for full-dose chemotherapy. Results showed that upfront dose-reduced chemotherapy was non-inferior to full-dose treatment and resulted in an improved toxicity profile, even in less frail patients.^17^ Thus, dose-reduced chemotherapy can improve QoL and physical functioning and decrease hospital admissions. Despite these promising results, upfront dose reduction is not yet widely adopted in Dutch daily practice and has not yet been studied in a phase III study or in chemotherapy schemes used in the Dutch setting.

### Study objectives

The primary objective of the DOSAGE study is to show that upfront dose-reduced chemotherapy in patients with metastatic colorectal cancer is non-inferior to full-dose treatment in terms of PFS with adaption of the treatment plan (monotherapy or doublet chemotherapy) based on expected risk of treatment toxicity for the individual patient. Secondary objectives are to investigate that upfront dose-reduced chemotherapy will lead to lower toxicity rates, better QoL and physical functioning, less dose reductions, treatment withdrawals and hospital admissions, a better overall survival (OS), a higher cumulative received dose and lower healthcare costs.

## Methods

### Study design

The DOSAGE study is an investigator-initiated, phase III, open-label, non-inferiority, randomised controlled clinical trial. The trial will enrol patients in 36 Dutch hospitals (2 academic and 34 peripheral hospitals). A list of all participating hospitals can be found at clinicaltrials.gov (NCT06275958).

### Patient population

Patients aged 70 years or older with colorectal cancer and metastasis without localised treatment options, eligible for first-line palliative chemotherapy at the discretion of the treating oncologist, will be included. Patients who received prior adjuvant chemotherapy in the year before inclusion, candidates for triplet therapy and those with a complete or incomplete dihydropyridine dehydrogenase deficiency or microsatellite instability-high colorectal cancer or with grade≥2 peripheral polyneuropathy are ineligible.

### Intervention

**Figure 1:**
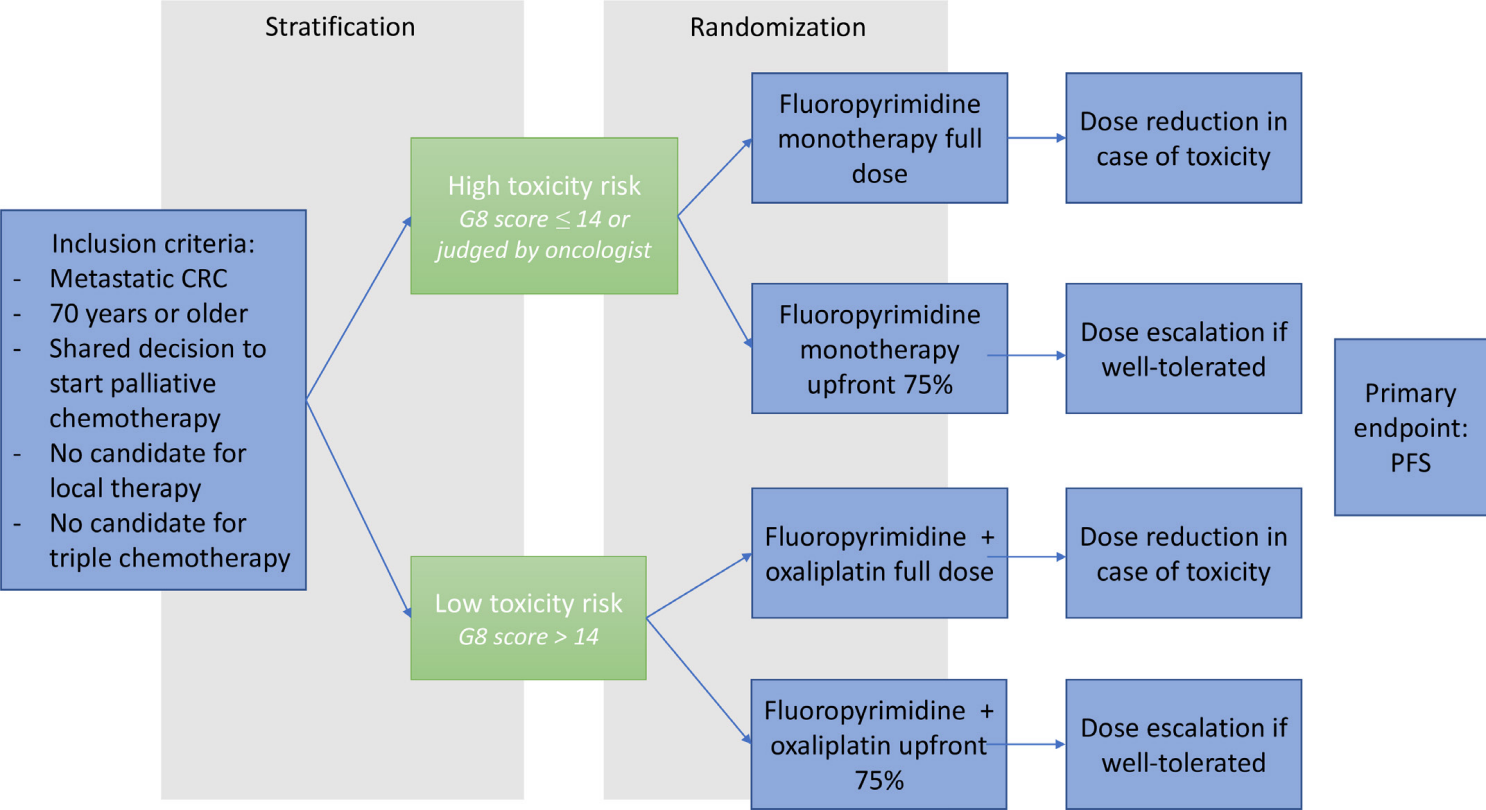
Schematic overview of study design. CRC, colorectal cancer; G8, Geriatric 8; PFS, progression-free survival.

Participants will be enrolled by their treating physician. All participants will first undergo geriatric screening by the G8 tool. Based on the G8, participants with a ‘low risk of toxicity’ (G8 Score of 15 or higher) will be stratified to doublet chemotherapy and those with a ‘high risk of toxicity’ (G8 Score of 14 or lower or judged as ‘high toxicity risk’ at the discretion of the treating oncologist) will be stratified to monotherapy (figure 1). Participants will then be randomised in a 1:1 ratio using block randomisation via the Castor electronic data capture (EDC) by the Clinical Research Center. Patients will be randomised between upfront dose-reduced chemotherapy or full-dose chemotherapy. In the dose-reduced intervention arm, dose escalation after two cycles is allowed, as explained in detail below. In the randomisation process, patients will be stratified by additional targeted therapy, tumour side and hospital.

### Fluoropyrimidine monotherapy

Patients classified as ‘high risk of toxicity’ will be randomised between monotherapy with a fluoropyrimidine in either full-dose or with an upfront reduction. The following dosing scheme is allowed in the full-dose arm:

- Capecitabine 1000 mg/m^2^ at day 1–14 (every 3 weeks) (see Discussion for rationale).

In the dose-reduced arm, patients receive an upfront 25% reduction (75% of full dose, 750 mg/m^2^).

### Doublet chemotherapy

Patients classified as low risk will be randomised between doublet chemotherapy with a fluoropyrimidine (physicians’ choice fluorouracil (5-FU) or capecitabine) and oxaliplatin in either full-dose, or with an upfront dose reduction. In the full-dose arm, the following dosing schemes are allowed:

- CAPOX: capecitabine 1000 mg/m^2^ at day 1–14 (every 3 weeks) and oxaliplatin 130 mg/m^2^ at day 1 (every 3 weeks).
- FOLFOX6: 5-FU 400 mg/m^2^ intravenous bolus at day 1 followed by 2400 mg/m^2^ in 46 hours (every 2 weeks), leucovorin 400 mg/m^2^ day 1 (every 2 weeks) and oxaliplatin 85 mg/m^2^ day 1 (every 2 weeks).

In the dose-reduced arm, patients receive an upfront 25% reduction (75% of full dose).

Addition of targeted treatment (bevacizumab or epidermal growth factor receptor (GFR) inhibition) is allowed. Patients with a moderate renal impairment (GFR 30–50 mL/min) will receive a 25% reduced starting dose of capecitabine in the full-dose arm and a 40% reduced starting dose in the dose-reduced arm.

### Treatment monitoring

After each treatment cycle, treatment tolerability (chemotherapy-related toxicity according to the Common Terminology Criteria for Adverse Events (CTCAE) V.5.0^18^) will be assessed. Patients in the upfront dose-reduced arm who have tolerated the first two cycles of their treatment well (no occurrence of grade 2–5 toxicity), can (but do not have to) receive dose-escalation to 100% at the discretion of the treating oncologist and after consultation with the patient. In both arms, toxicity may allow for further dose reductions: if patients experience either grade 3–4 toxicity or unacceptable grade 1–2 toxicity that influences QoL, the oncologist can reduce the dose by 25%. The minimal dose is 50% of full dose: if further (unacceptable) toxicity occurs, the treatment should be discontinued. For neuropathy, in case of grade 2 toxicity, oxaliplatin dose is reduced with 25%. In case of grade 3 neuropathy, oxaliplatin is definitively stopped. After 18 weeks of doublet chemotherapy, maintenance therapy with capecitabine or 5-FU (with or without bevacizumab) can be started at the discretion of the treating oncologist. Chemotherapy treatment will be continued until progression, unacceptable toxicity or a patients’ or clinicians’ decision to stop. If patients develop progression or discontinue first-line treatment due to other reasons (such as toxicity), the study will end and any further treatment lines are allowed by the discretion of the oncologist.

### Study procedures

Apart from the G8 that is performed to stratify patients between monotherapy and doublet therapy, participants will also undergo a GA. The results from the GA will be used to characterise the study population and find predictors of poor outcomes. The GA will comprise the EuroQol-5D (EQ-5D)^19^ and European Organisation for Research and Treatment of Cancer (EORTC) Core QLQ-C30 questionnaires for QoL, the Katz Activities of Daily Living (ADL),^20^ Lawton Instrumental ADL (IADL)^21^ and home and informal care for physical functioning, the Modified Telephone Interview for Cognitive Status for cognition,^22^ Mini Nutritional Assessment for malnutrition^23^ and Patient Health Questionnaire-2 for mood^24^ (table 1). The GA will be conducted via telephone calls to increase feasibility. Performing a telephone-based GA has proven to be feasible in previous studies with older adults.^25 26^ Comorbidity (assessed with the Charlson Comorbidity Index^27^) and polypharmacy (at least five different types of medication) will be obtained from the medical charts. If the GA identifies geriatric deficits, the treating oncologist will be informed and GA-based interventions will be at their discretion.

**Table 1:** Overview of study procedures

	Screening (T<0)	Prerandomisation	Randomisation (T=0)	Before and after every cycle, until progression	Each 8–12 weeks	After 1 month	After 3 months	After 6 months	After 9 months	After 12 months	End of study
Informed consent		E									
Geriatric-8		E									
Inclusion/exclusion	X										
Patient/tumour characteristics	X										
Laboratory analysis*	X			X							
Toxicity				X							
Radiologic evaluation†	X				X						
Dose reductions				X							
Cycles, n				X							
Hospitalisations				X							
Survival					X						X
EQ-5D‡		E				E	E	E	E	E	
EORTC Core QLQ-C30‡		E				E	E	E	E	E	
Katz ADL‡		E				E	E	E	E	E	
Lawton IADL‡		E				E	E	E	E	E	
Home and informal care‡		E				E	E	E	E	E	
MNA		E									
TICS-M		E									
PHQ-2		E									

Window for questionnaires: ±4 weeks.

X=procedures are standard of care. E=extra procedure in study context.

* Laboratory analyses should at least include total blood count, kidney and liver function.

† Radiologic evaluation: performed according to local standard practice (CT scan, positron emission tomography (PET) scan and/or MRI scan).

‡ Follow-up questionnaires will be performed until disease progression or until 12 months after randomisation in case of no disease progression.

ADLactivities of daily livingEORTCEuropean Organisation for Research and Treatment of CancerEQ-5DEuroQol-5DIADLInstrumental Activities of Daily LivingMNAMini Nutritional AssessmentNnumberPHQ-2Patient Health Questionnaire-2TICS-MModified Telephone Interview for Cognitive Status

After 1 3, 6, 9 and 12 months, alive patients will be invited to complete four short questionnaires on QoL (EORTC QLQ-C30 and EQ-5D) and physical functioning (Lawton IADL and Katz ADL) plus questions about home and informal care. These questionnaires can be answered by telephone or sent by email (whatever the patient prefers) to optimise follow-up data.

### Outcome measures

The *primary outcome* of the study is PFS, defined as time from randomisation until either radiological or clinical progression or death due to any cause, whichever occurs first. Timepoints of measurements are presented in table 1. In case of clinical progression as judged by the local principal investigator, local investigators should explain the base of clinical progression. *Secondary endpoints* include QoL (measured by the EQ-5D and EORTC Core QLQ-C30 questionnaires after 1, 3, 6, 9 and 12 months), physical functioning (measured with the Katz ADL, the Lawton IADL and home and informal care after 1, 3, 6, 9 and 12 months), grade≥3 chemotherapy-related toxicity according to the CTCAE during study treatment, dose reductions during study treatment, number of treatment cycles, unplanned hospital admissions within the first year, OS, cumulative received dose and cost-effectiveness in the first year. Predictive geriatric markers of poor outcomes will be identified using the baseline GA.

### Observational cohort for patients who do not want to undergo randomisation

Previous studies have shown that inclusion of older adults with cancer can be challenging.^28^ One reason is that patients or their physicians may strongly prefer a particular treatment strategy and therefore do not want to undergo randomisation. To still capture all eligible patients and assess differences in tumour and patient characteristics between participants and non-participants, we will register patients in an observational study arm. Patients need to provide written consent for the observational cohort but will not be randomised. They will be invited to complete the G8, Katz ADL, Lawton IADL and EQ-5D and questions about living status, previous falls and delirium at baseline and will receive one follow-up request at 3 months for the EQ-5D, Katz ADL, Lawton IADL.

### Data collection, management and monitoring

Clinical data will be extracted from electronic health records and collected on electronic case report forms designed for this study. Castor EDC, an EDC system, is used for data entry and storage. On site monitoring is organised according to the Netherlands Federation of University Medical Centers guideline ‘Quality assurance of research involving human subjects’^29^ by dedicated monitors from the Leiden University Medical Center. As the study compares two frequently used and well-characterised chemotherapy regimens conform clinical practice, the study is classified as low risk and does not require a Data and Safety Monitoring Board or interim analysis.

#### Ethics and dissemination

This trial has received ethical approval by the ethical committee Leiden Den Haag Delft (P24.018) and will be approved by the Institutional Ethical Committee of the participating institutions. All participants will provide written informed consent and the study will adhere to the principles outlined in the Declaration of Helsinki. The results will be disseminated in peer-reviewed scientific journals.

#### Statistics: sample size and power calculations

Based on previous studies in older adults with metastatic colorectal cancer (table 2), the study assumes non-inferiority of the intervention arm with a median PFS of 8 months. We determined a non-inferiority margin of 8 weeks based on previous trials^16 17^ and extensive consultation with seven primary investigators from participating hospitals and the Dutch colorectal patient foundation, who all considered this an acceptable upper margin for non-inferiority.

**Table 2:** Median PFS in previous trials with older adults

Trial	Patient population	Median PFS
MRFOCUS2^15^	‘Non-fit’ older or frail mCRC patients receiving upfront dose reduction	Median PFS was 3.5, 5.8, 5.2 and 5.8 months in the 5-FU monotherapy, FOLFOX, capecitabine monotherapy and CAPOX groups, respectively; a reasonable estimate for all patients is 5 months
AVEX^33^	Older mCRC patients, no candidate for oxaliplatin or irinotecan	Median PFS 9.1 months for capecitabine+bevacizumab, 5.1 months for capecitabine monotherapy.
NORDIC9^16^	‘Non-fit’ older mCRC patients	Median PFS 5.1 months for full-dose S-1, 6.2 months for reduced-dose oxaliplatin+S-1. 25% received bevacizumab.
SALTO^34^	mCRC patients with median age 73 years (not specifically older patients)	Median PFS 8.2 months for capecitabine monotherapy, 8.4 months for S-1 monotherapy.

AVEXAVastin in the Elderly with XelodaCAPOXcapecitabine plus oxaliplatinFOLFOXfolinic acid, fluorouracil, and oxaliplatin5-FUFluorouracilmCRC, metastatic colorectal cancerPFSprogression-free survival

A non-inferiority logrank test with an overall sample size of 528 subjects (264 in the reference group and 264 in the treatment group) achieves 80.0% power at a 5% significance level to detect an equivalence HR of 1.25 when the actual HR is an equivalence HR of 1 and the reference group hazard rate is 0.12. The study will last for 48 time periods of which subject accrual entry occurs in the first 36 time periods. The accrual pattern across time periods is uniform (all periods equal). Accounting for an expected dropout rate of 10%, 587 patients in total are required.

### Statistics: analysis

Both per-protocol and an intention-to-treat analysis will be conducted. Patients will be grouped according to the treatment they were randomised to receive. Descriptive characteristics of patients will be reported using frequencies and proportions for categorical data and means and SD for continuous data.

*Primary outcome* PFS will be estimated from randomisation by using Kaplan-Meier’s methodology and Cox regression models. Non-inferiority of the dose-reduced arm can be claimed if the upper limit of the CI of the HR is below 1.25. Univariable and multivariable Cox proportional hazard regression models will be estimated to investigate the effect of age, comorbidity, the geriatric domains, cumulative received dose and tumour characteristics on survival. To study the effect of treatment among risk groups, an interaction term between will be included in the model together with additional targeted therapy and centre. As an exploratory planned subgroup analysis, PFS will be stratified based on molecular pathologic features.

#### Secondary outcomes

A logistic regression model will be estimated to study the effect of groups on chemotherapy-related toxicity; the same covariates as discussed before will be included. Due to the presence of repeated measures for QoL and physical functioning, mixed models to investigate the relation between the outcomes and arms will be used. An interaction term between time and arms will be added to the model to quantify the effect of time. The cumulative dose received by patients in each arm (divided by BSA) will be reported, and means will be compared using the t-test. Finally, we will investigate which geriatric domains (assessed by the GA) are associated with toxicity, QoL and physical functioning.

For cost-effectiveness, a cost-utility analysis will be performed (‘costs per quality-adjusted life year (QALY)’) from a societal perspective and with a life-long time horizon, in accordance with the Dutch guidelines for economic evaluations in healthcare.^30^ Costs will include the chemotherapy and other hospital costs (assessed from study registrations) and home and informal care (assessed using patient questionnaires at 1, 3, 6, 9 and 12 months). No impact on productivity costs will be assumed, because of the patients’ age. QALYs will be calculated using the Dutch tariff for the five-level EQ-5D questionnaires filled out by patients.^19^ Mathematical modelling will be used to extrapolate outcomes to a life-time horizon. In the analysis, incremental costs and QALYs will be compared using net-benefit analysis, according to intention to treat.^31^

#### Trial status

Expected date of first inclusion is July 2024 and the study is expected to end in January 2029.

### Patient and public involvement

The Dutch patient advocate foundation *Stichting Darmkanker* is a coinitiator of this study. The foundation has actively participated in the first two phases of the study (defining the research question and writing a study proposal) and has given extensive feedback on the current protocol and design and the study burden. The foundation will remain actively involved in the last two study phases as well (data collection and analyses/dissemination). A member of the foundation is an active member in our research group and money has been budgeted for their participation.

## Discussion

The DOSAGE study will improve the evidence on the effect of dose-reduced chemotherapy on survival and treatment outcomes in older patients. Additionally, it will provide new insights into the use of the G8 tool to choose between doublet chemotherapy or monotherapy, enabling tailor-made treatment decisions based on the individual health status of patients. This approach, together with upfront dose reduction, will lead to a more individualised treatment strategy and can potentially reduce chemotherapy-related toxicity and hospitalisations, improve QoL and physical functioning, while maintaining treatment efficacy. This innovative trial design, which incorporates toxicity risk stratification, may serve as an example for future research in this understudied and growing population.

In comparison with some previous studies, we selected 1000 mg/m^2^ as full dose of capecitabine instead of 1250 mg/m^2^.^32^ The rationale behind this choice is that we believe frail older adults should not be exposed to a dose of 1250 mg/m^2^. In Dutch daily practice, 1000 mg/m^2^ is the most commonly used dosage for older adults in the Netherlands. Although some studies with capecitabine were performed with the dosage of 1250 mg/m^2^ as full dose, the AVastin in the Elderly with Xeloda (AVEX) trial, randomising older patients between capecitabine plus bevacizumab or capecitabine alone,^33^ and the CAIRO7 trial, designed for older and frail patients with unresectable liver metastases of colorectal cancer, also used 1000 mg/m^2^ as full dose. A full dose of 1000 mg/m^2^ for older adults was also used in the SALTO trial, comparing S-1 and capecitabine as first-line treatment for metastatic colorectal cancer.^34^ The Dutch Colorectal Cancer Group agreed to the definition of 1000 mg/m^2^ as standard dose, further supporting this decision.

Recruiting older patients for clinical trials can be challenging, but with 3.500 older patients diagnosed with metastatic colorectal cancer in the Netherlands each year^35^ and the involvement of 36 participating hospitals, we anticipate successful enrolment. Previous Dutch studies supported by the Dutch Colorectal Cancer Group, such as the CAIRO,^36^ CAIRO2^37^ and CAIRO5^38^ trials, were also able to include large numbers of Dutch patients with metastatic colorectal cancer (530, 755 and 530 patients with metastatic colorectal cancer, respectively). The DOSAGE is designed to closely resemble daily practice and apart from answering questionnaires over the phone, participants do not need to have additional biopsies or blood withdrawals taken. Hence, the burden and risks for patients are minimal, which will hopefully enhance patient participation.

Since the G8 has a very high sensitivity, but a lower specificity,^13 39^ a trial risk is the potential misclassification of a small subset of fit patients as having high risk for toxicity based on the G8 screening, leading to stratification in the monotherapy arm and a possibility of undertreatment. However, these patients can still receive second-line doublet chemotherapy in case of progression if they maintained robust during monotherapy treatment. Since these patients would have already met the primary endpoint (PFS) by progressing on first-line treatment, this does not bias study outcomes. Previous studies demonstrated that this approach (sequential use of chemotherapy instead of doublet chemotherapy) does not jeopardise OS, making this a safe treatment option.^36 40^

The concern of undertreatment due to upfront dose reduction is addressed by allowing dose escalation based on treatment tolerability. However, the MRFOCUS2 trial showed that, in older patients treated with upfront dose-reduced chemotherapy, dose escalation was only possible in 20%–30% and only 14% could sustain a dose escalation to 12 weeks, while 50% needed a further dose reduction or stopped.^15^ These data suggest that risk of undertreatment in the dose-reduced arm is minimal.

In conclusion, the DOSAGE trial will provide new evidence regarding the effect of dose-reduced chemotherapy on outcomes and the use of the G8 tool in choosing between doublet chemotherapy or monotherapy. The design can serve as an example for trials studying upfront dose-reduced chemotherapy in other tumour types. Moreover, the results will contribute to a more individualised approach in older patients with metastatic colorectal cancer and improved treatment outcomes for this large and growing population.
