# Supplementary material for: DOSAGE study: protocol for a phase III non-inferiority randomised trial investigating dose-reduced chemotherapy for advanced colorectal cancer in older patients
Source: BMJ Open. 2024 Aug 13;14(8):e089882. doi: 10.1136/bmjopen-2024-089882 (PMC11331880; doi:10.1136/bmjopen-2024-089882)
Supplement: Supplementary file 1 [file bmjopen-2024-089882.reviewer_comments.pdf]

PEER REVIEW HISTORY

BMJ Open publishes all reviews undertaken for accepted manuscripts. Reviewers are asked to complete a checklist review form (**http://bmjopen.bmj.com/site/about/resources/checklist.pdf**) and are provided with free text boxes to elaborate on their assessment. These free text comments are reproduced below.

ARTICLE DETAILS

**TITLE (PROVISIONAL)**	The DOSAGE study: Protocol for a Phase III Non-inferiority Randomized Trial investigating Dose-reduced Chemotherapy for Advanced Colorectal Cancer in Older Patients
**AUTHORS**	Baltussen, Joosje; van den Bos, Frederiek; Slingerland, Marije; Binda, Trishika; Liefers, Gerrit-Jan; van den Hout, Wilbert; Fiocco, Marta; Verschoor, Arjan J.; Cloos - van Balen, Marissa; Holterhues, Cynthia; Houtsma, Danny; Jochems, Anouk; Spierings, Leontine E.A.M.M.; van Bodegom - Vos, Leti; Mooijaart, Simon; Gelderblom, Hans; Speetjens, Frank; de Glas, Nienke A.; Portielje, Johanneke

This article was not externally reviewed at BMJ Open. Protocols that have been independently assessed prior to submission to BMJ Open are usually fast-tracked to publication on the grounds that further substantial changes will not be possible. This independent assessment will usually be external, independent review for both a substantial grant award from a non-commercial or government funder and ethics approval.
